# Associations between healthy lifestyle and mortality across different social environments: a study among adults with frailty from the UK Biobank

**DOI:** 10.1093/eurpub/ckae003

**Published:** 2024-01-30

**Authors:** Junhan Tang, Yanan Ma, Emiel O Hoogendijk, Jie Chen, Jirong Yue, Chenkai Wu

**Affiliations:** Global Health Research Center, Duke Kunshan University, Kunshan, Jiangsu, China; Department of Biostatistics and Epidemiology, School of Public Health, China Medical University, Shenyang, Liaoning, China; Department of Epidemiology & Data Science, Amsterdam Public Health Research Institute, Amsterdam UMC—location VU University Medical Center, Amsterdam, The Netherlands; Center for Global Health, Zhejiang University School of Medicine, Hangzhou, Zhejiang, China; Department of Geriatrics and National Clinical Research Center for Geriatrics, West China Hospital, Sichuan University, Chengdu, Sichuan, China; Global Health Research Center, Duke Kunshan University, Kunshan, Jiangsu, China

## Abstract

**Background:**

Among people living with frailty, adherence to a healthy lifestyle may be a low-cost and effective strategy to decrease frailty-induced health risks across different social environments.

**Methods:**

We included 15 594 frail participants at baseline from the UK Biobank study. We used four lifestyle factors to create a composite healthy lifestyle score and 17 social factors to construct a polysocial score. We classified the lifestyle score into two levels (unhealthy and healthy) and the polysocial score into three levels (low, intermediate and high). We used Cox regression to determine the association of each lifestyle factor and lifestyle score with all-cause mortality, respectively. We also examined the associations across polysocial score categories. We evaluated the joint association of the lifestyle score and the categorical polysocial score with all-cause mortality.

**Results:**

During up to 14.41 follow-up years, we documented 3098 all-cause deaths. After multivariable adjustment, we found a significant association between not smoking and adequate physical activity with all-cause mortality across polysocial score categories, respectively. We also found a significant association between a healthy diet and all-cause mortality among frail participants living in an intermediate social environment. A healthy lifestyle was associated with a lower all-cause mortality risk across polysocial score categories, especially among those with a low polysocial score.

**Conclusions:**

Adherence to a healthy lifestyle, particularly not smoking, adequate physical activity and a healthy diet, may provide a feasible solution to decreasing mortality risk among frail adults across different social environments, especially for those in the socially disadvantaged group.

## Introduction

Physical frailty is a prevalent aging-related geriatric syndrome characterized by a decreased reserve capacity to cope with stressors.^1^ Over the past two decades, research on frailty has proliferated, focusing on the development and validation of measurement tools, quantification of the prevalence, identification of risk factors and investigation of its impact on adverse outcomes.^2^ Frail older adults are at high risk of hospitalization, disability and mortality.^3–5^ Interventions aiming to reserve the state of frailty have become increasingly available.^6^^,^^7^ However, effective strategies are generally still lacking, due to the complicated etiology of frailty and the heterogeneity among the frail population.^8^ Intervention efforts have focused on improving functions and alleviating symptoms instead of targeting frailty-causing biology and treating the syndrome per se. Frail individuals are a socioeconomically, behaviorally and clinically diverse group. Therefore, identifying socioeconomic, lifestyle and health features that could mitigate frailty-induced health risks and promote resilience is critical for guiding patient-centered management of frailty.

Healthy lifestyles, which represent a group of modifiable factors, are associated with various adverse outcomes among adults, regardless of age.^9–12^ Mounting evidence suggests that adhering to a healthy lifestyle could reduce the risk of mortality, disability and hospitalization among older adults.^13–15^ However, whether these lifestyle factors remain predictive of outcomes among people with frailty is unknown. Additionally, people with frailty are an economically heterogeneous population; some live in a desirable social environment with a stable economic condition and high social support, while others suffer from unfavorable social conditions. Clear evidence shows a higher prevalence of unhealthy behaviors among persons with a low socio-economic position, receiving inadequate social support and living in poor neighborhoods.^16^^,^^17^ A disadvantaged social position might attenuate the benefits of adhering to a healthy lifestyle; the association between lifestyles and health outcomes might not be universal in different social environments.

The aim of the present study was 3-fold. First, we examined whether adherence to a healthy lifestyle, including not smoking, no excessive alcohol consumption, adequate physical activity and a healthy diet, was associated with a lower mortality among frail persons. Second, we examined the association between lifestyle factors and all-cause mortality in different social environments. Third, we evaluated the joint association of lifestyles and social environment with all-cause mortality. We adopted the polysocial score approach,^18–21^ a novel tool developed to capture the aggregate effects of social factors, to measure the social environment. The findings of this work may contribute to clinical practice guidelines on the management of frailty, including the development of patient-centered clinical care plans.

## Methods

### Data source

We used data from the UK Biobank, a prospective cohort study with >500 000 participants (40–69 years) between 2006 and 2010 from 22 assessment centers across England, Wales and Scotland. In practice, participants who were outside the age limit were also included (37–73 years). Participants were invited to attend the closest assessment center to complete a self-administered touch-screen questionnaire, a nurse-led, face-to-face interview, physical measurements and bio-specimen sample collection. The UK Biobank enables the follow-up of medical and health-related records. All participants signed informed consent for data collection, analysis and linkage. The North West-Haydock Research Ethics Committee (REC reference: 16/NW/0274) granted ethical approval to the UK Biobank. Details of the study design and data collection are documented elsewhere.^22^ The present study is part of the UK Biobank project 51450.

### Analytic sample

We leveraged data from frail participants at baseline. Frailty was assessed by the physical frailty phenotype (PFP) approach.^23^ We used a modified version adapted to the available questions and measurements in the UK Biobank.^24^ Five criteria were included: slowness, weakness, exhaustion, inactivity and shrinking. Participants were classified as frail if they met three or more criteria. Operational definitions of frailty criteria are in Supplementary table S1. We excluded participants who were not frail (*n* = 486 322). Individuals with missing data for lifestyle factors (*n* = 496) were excluded. The analytic sample included 15 594 participants with frailty (Supplementary figure S1).

### Mortality

The primary outcome was all-cause mortality. Death information was obtained through death certificates available in the National Health Service (NHS) Information Center for participants from England and Wales and the NHS Central Register, National Records of Scotland for participants from Scotland. Details of the linkage procedure can be found at http://content.digital.nhs.uk/services.^25^ Death data were available up to 26 October 2021; follow-up for death events was censored on this date or the date of death if this occurred earlier.

### Lifestyle factors

We included four lifestyle factors: smoking, alcohol consumption, physical activity and diet. We classified smoking into not smoking and current/previous smoking.^16^ Participants were asked about the consumption amount of wine, beer, spirit and other alcoholic drinks. We used the frequency and volume of alcohol consumption to calculate daily alcohol consumption.^26^ According to the dietary guidelines in the UK,^27^ we considered daily consumption of ≤8 g for female and ≤16 g for male as no excessive alcohol consumption, and otherwise an unhealthy level. For physical activity, we calculated a total weekly metabolic equivalent time period for four activities: walking for pleasure, heavy DIY, strenuous sports and other exercises.^28^ We classified participants into three tertiles according to the total weekly metabolic equivalent time period, and considered the top layer as adequate physical activity,^19^ and the other layers as an unhealthy level. We measured dietary quality using a dietary recommendation for cardiovascular diseases.^29^ A healthy diet was defined as satisfying no less than five items of the food recommendation,^16^ while an unhealthy diet was defined as meeting less than or equal to four items. The detailed descriptions of the four lifestyle factors are provided in Supplementary table S2. Following previous investigations using the UK Biobank data, we assigned 1 point for a healthy level and 0 points for an unhealthy level for each lifestyle factor and constructed a sum score, ranging from 0 (unhealthiest) to 4 (healthiest).^16^^,^^29^

### Social factors

Guided by previous literature,^18^ we included 17 social factors from three categories: socioeconomic status, psychosocial factors and neighborhood and living environment. Socioeconomic status included the highest education level, education score and employment status. Psychosocial factors included living situation, social support, social activity, social isolation, emotional distress and psychiatric disorder. Neighborhood and living environment included the Townsend deprivation index, crime rate, housing quality, accommodation ownership, type of house, and remoteness of greenspace, bluespace and natural land. Each social factor was classified into a favorable level and a reference level.

For each social factor, the detailed definitions of a favorable and reference level were provided in Supplementary table S3, and we assigned 1 point for a favorable level and 0 points for a reference level. We imputed the missing values using the average of non-missing social factors for each individual. We constructed a polysocial score using the sum score.^18^ We classified the polysocial score into three categories: low (lowest quartile), intermediate (quartiles 2–3) and high (highest quartile).

### Covariates

Covariates included age, classified into <50 years, 50–59.9 years and ≥60 years, sex and race/ethnicity categorized as Whites or Others (a common strategy to classify race/ethnicity in UK Biobank).

### Statistical analysis

We described the study participants by polysocial score (high, intermediate and low) using means and SDs for continuous variables and counts and percentages for categorical variables. Comparisons were made by polysocial score using analysis of variance for continuous variables and the chi-square test for categorical variables. Only 2487 (15.9%), 1101 (7.1%) and 100 (0.6%) participants had a healthy lifestyle score of 0, 3 and 4 points, respectively. We, therefore, created a binary healthy lifestyle score. A score of 0–1 indicated an unhealthy level, while a score of 2–4 indicated a healthy level. This is a common strategy to categorize the composite healthy lifestyle score in UK Biobank.^16^

We used the Kaplan–Meier approach to compare the survival functions between participants with an unhealthy and healthy level of lifestyle in the low, intermediate and high polysocial score categories, respectively. We calculated the incident mortality rates by each lifestyle factor and the composite lifestyle score. We then used Cox models to identify the association of each lifestyle factor and the composite healthy lifestyle score with mortality, respectively. Age, sex and race/ethnicity were included in the adjusted models. We further conducted stratified analyses by polysocial score category (high, intermediate and low) to assess the association of each lifestyle factor and the composite healthy lifestyle score with all-cause mortality, respectively. Within each polysocial score category, we set the unhealthy level as the reference group. We examined the additive and multiplicative interaction between the polysocial score and each lifestyle factor, respectively. We also examined the interaction between the polysocial score and the composite healthy lifestyle score. To evaluate the joint effect of the composite healthy lifestyle score and the polysocial score on all-cause mortality, we classified participants into six categories according to the lifestyle score (unhealthy and healthy) and the polysocial score (low, intermediate and high), with individuals having an unhealthy lifestyle and a low polysocial score as the reference level.

We conducted several sensitivity analyses. First, we created another healthy lifestyle score: a score of 0–1 indicated an unhealthy level; a score of 2 indicated an intermediate level and a score of 3–4 showed a healthy level. Using the Kaplan–Meier approach, we compared the survival functions among participants with an unhealthy, intermediate and healthy level of lifestyle across polysocial score categories. In addition, we repeated the main analyses by age (≥60 or <60 years) and sex.

All tests were two-sided with a significance level of 0.05. All statistical analyses were conducted in R 4.1.2.

## Results

### Sample characteristics

The average age of the 15 594 frail participants was 57.9 years (SD = 7.6 years), 48.2% were ≥60 years, 62.7% were female, and 87.1% were White participants (table 1). Compared to participants with a low and intermediate polysocial score, those in the high polysocial score category were more likely to be female, older and White. They also had higher prevalence of a healthy level of smoking and physical activity, and a higher composite healthy lifestyle score. The polysocial score ranged from 0 to 16, with a mean of 7.6 (SD = 2.6). The distribution of the polysocial score was approximately normal (Supplementary figure S2). Of all participants, 5217 (33.5%), 6513 (41.8%) and 3864 (24.8%) had a low, intermediate and high polysocial score, respectively.

**Table 1:** Socio-demographic and lifestyle characteristics by polysocial score (high, intermediate and low) among frail participants

**Characteristics** ^a^		Polysocial score		
	Total sample	High	Intermediate	Low
	*N* = 15 594 (100.0%)	*N* = 3864 (24.8%)	*N* = 6513 (41.8%)	*N* = 5217 (33.5%)
Female (*N*, %)	9772 (62.7)	2650 (68.6)	4155 (63.8)	2967 (56.9)
Age (mean, SD)	57.9 (7.6)	58.3 (7.4)	57.9 (7.8)	57.6 (7.6)
Age category (*N*, %)				
<50 years	2644 (17.0)	583 (15.1)	1148 (17.6)	913 (17.5)
50–59.9 years	5431 (34.8)	1356 (35.1)	2141 (32.9)	1934 (37.1)
≥60 years	7519 (48.2)	1925 (49.8)	3224 (49.5)	2370 (45.4)
Race/ethnicity (*N*, %)				
Whites	13 587 (87.1)	3640 (94.2)	5650 (86.7)	4297 (82.4)
Others	1922 (12.3)	210 (5.4)	822 (12.6)	890 (17.1)
Unclear	85 (0.5)	14 (0.4)	41 (0.6)	30 (0.6)
Smoking (*N*, %)				
Unhealthy level	8341 (53.5)	1804 (46.7)	3440 (52.8)	3097 (59.4)
Healthy level	7253 (46.5)	2060 (53.3)	3073 (47.2)	2120 (40.6)
Alcohol consumption (*N*, %)				
Unhealthy level	4370 (28.0)	1252 (32.4)	1826 (28.0)	1292 (24.8)
Healthy level	11 224 (72.0)	2612 (67.6)	4687 (72.0)	3925 (75.2)
Physical activity (*N*, %)				
Unhealthy level	14 652 (94.0)	3588 (92.9)	6109 (93.8)	4955 (95.0)
Healthy level	942 (6.0)	276 (7.1)	404 (6.2)	262 (5.0)
Diet (*N*, %)				
Unhealthy level	13 646 (87.5)	3349 (86.7)	5710 (87.7)	4587 (87.9)
Healthy level	1948 (12.5)	515 (13.3)	803 (12.3)	630 (12.1)
Healthy lifestyle score (*N*, %)				
0	2487 (15.9)	579 (15.0)	1056 (16.2)	852 (16.3)
1	6148 (39.4)	1470 (38.0)	2494 (38.3)	2184 (41.9)
2	5758 (36.9)	1481 (38.3)	2458 (37.7)	1819 (34.9)
3 and 4	1201 (7.7)	334 (8.6)	505 (7.8)	362 (6.9)

a *P*-values were calculated using analysis of variance for continuous variables and chi-square test for categorical variables. All *P*-values were less than .01, except for diet (*P* = .18).

### Association of lifestyles with mortality

Frail participants with a healthy level of smoking, alcohol consumption, physical activity and diet had a 10.5, 3.0, 3.5 and 1.7 per 1000 person-years lower incidence rate of mortality than those with an unhealthy level, respectively (table 2). After multivariable adjustment, participants with a healthy level of smoking, physical activity and diet had a 40, 33 and 15% lower hazard of mortality than their counterparts, respectively. The difference between persons with a healthy and unhealthy level of alcohol use did not persist in the adjusted model.

**Table 2:** Association of lifestyle factors with all-cause mortality (*n* = 15 594)

Lifestyle factors	All-cause mortality rate per 1000 PYs	HR (95% CI)	
		Unadjusted	**Adjusted** ^a^
**Smoking**			
Unhealthy	20.3	Ref.	Ref.
Healthy	9.8	0.48 (0.44–0.52)	0.60 (0.56–0.65)
**Alcohol consumption**			
Unhealthy	17.4	Ref.	Ref.
Healthy	14.4	0.82 (0.76–0.89)	0.99 (0.92–1.07)
**Physical activity**			
Unhealthy	15.4	Ref.	Ref.
Healthy	11.9	0.77 (0.66–0.91)	0.67 (0.57–0.79)
**Diet**			
Unhealthy	15.4	Ref.	Ref.
Healthy	13.7	0.89 (0.79–0.99)	0.85 (0.76–0.95)
**Healthy lifestyle score**			
Continuous		0.72 (0.69–0.75)	0.81 (0.77–0.84)
Categorical			
0–1	19.3	Ref.	Ref.
2–4	10.5	0.54 (0.50–0.59)	0.66 (0.61–0.71)

PYs, person-years; HR, Hazard Ratio; CI, Confidence Interval.

a Adjusted for age, sex, and ethnicity.

After adjusting for age, sex and race/ethnicity, a one-point higher continuous healthy lifestyle score was associated with a 19% lower hazard of mortality. When modeled as a composite healthy lifestyle score (healthy vs. unhealthy), individuals with a healthy level of the composite lifestyle score had an 8.8 per 1000 person-years lower incidence rate of all-cause mortality than those with an unhealthy level. The hazard of mortality among participants in the healthy level was 34% (95% CI = 29–39%) lower than those in the unhealthy level. We found similar patterns when examining the association of each lifestyle factor and the composite healthy lifestyle score with mortality by age (at least or less than 60 years) or sex (Supplementary tables S4 and S5).

### Association between lifestyles and mortality by polysocial score

**Figure 1:**
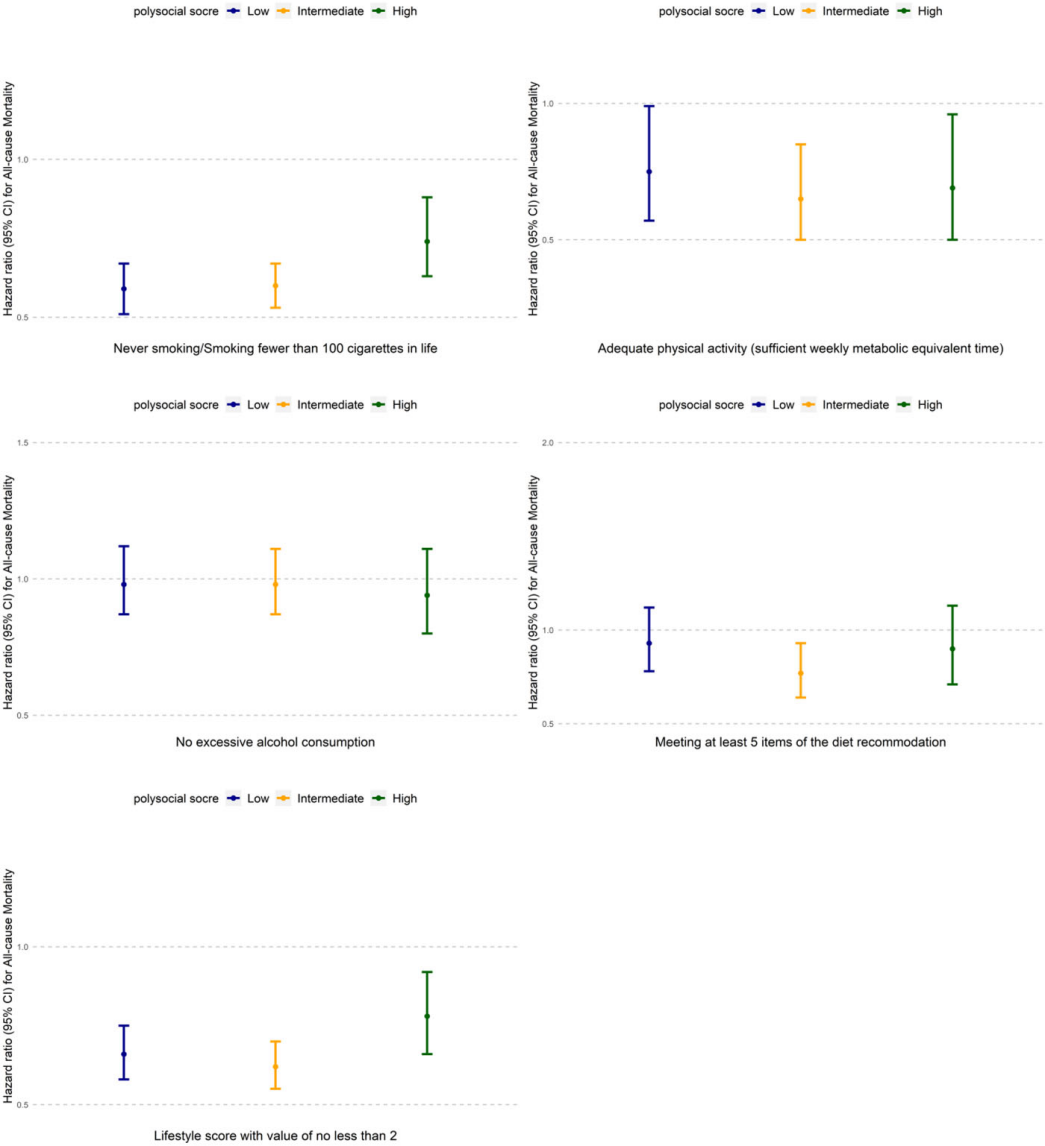
Association of lifestyle factors with all-cause mortality across polysocial score categories (*n* = 15 594). Notes: CI, Confidence Interval. Hazard ratios were adjusted for age, sex, and ethnicity. Additive and multiplicative interaction was assessed using the hazard ratio for the product interaction between each lifestyle factor (smoking, alcohol consumption, physical activity, diet, and the composite healthy lifestyle score) and the categorical polysocial score. For the composite healthy lifestyle score, the unhealthy level represented 0 and 1, and the healthy level represented 2, 3 and 4. The *P*-value for additive or multiplicativive interaction between never smoking/smoking fewer than 100 cigarettes in life and a high polysocial score was 0.04.

Figure 1 presents the associations of lifestyle factors with mortality in each polysocial score category (low, intermediate and high). After multivariable adjustment, a healthy level of smoking was associated with a lower hazard of mortality among participants in each polysocial score category (hazard ratios [HRs] = 0.59, 0.60 and 0.74). A healthy level of physical activity was associated with a lower hazard of mortality among participants in each polysocial score group (HRs = 0.75, 0.65 and 0.69). A healthy level of diet was associated with a lower hazard of mortality only among participants with an intermediate polysocial score (HR = 0.77). We observed both positively additive and multiplicative interactions between a healthy level of smoking and a high polysocial score. No significant additive or multiplicative interaction was found between the polysocial score and other lifestyle factors. A healthy level of the composite healthy lifestyle score was associated with a lower adjusted hazard of mortality among participants with a low, intermediate and high polysocial score (HRs = 0.66, 0.62 and 0.78). We did not find any additive or multiplicative interaction between the polysocial score and the composite healthy lifestyle score. Results were similar when we stratified the analyses by age (at least or less than 60 years) or sex (Supplementary figures S3–S6).

### Healthy lifestyle, polysocial score and mortality

In each of the three polysocial score categories, participants with a healthy level of the composite healthy lifestyle score (score: 2–4) had a higher survival than those with an unhealthy level (score: 0–1; Supplementary figure S7). The difference in survival between the healthy level and the unhealthy level of the composite healthy lifestyle score was smaller among participants with an intermediate polysocial score than those in the low polysocial score category; the difference further decreased among those with a high polysocial score. We found similar patterns when repeating the analyses among participants aged 60 years or above (Supplementary figure S8). The patterns of the survival functions for mortality did not significantly change when using the three-level healthy lifestyle score (Supplementary figure S9).

**Figure 2:**
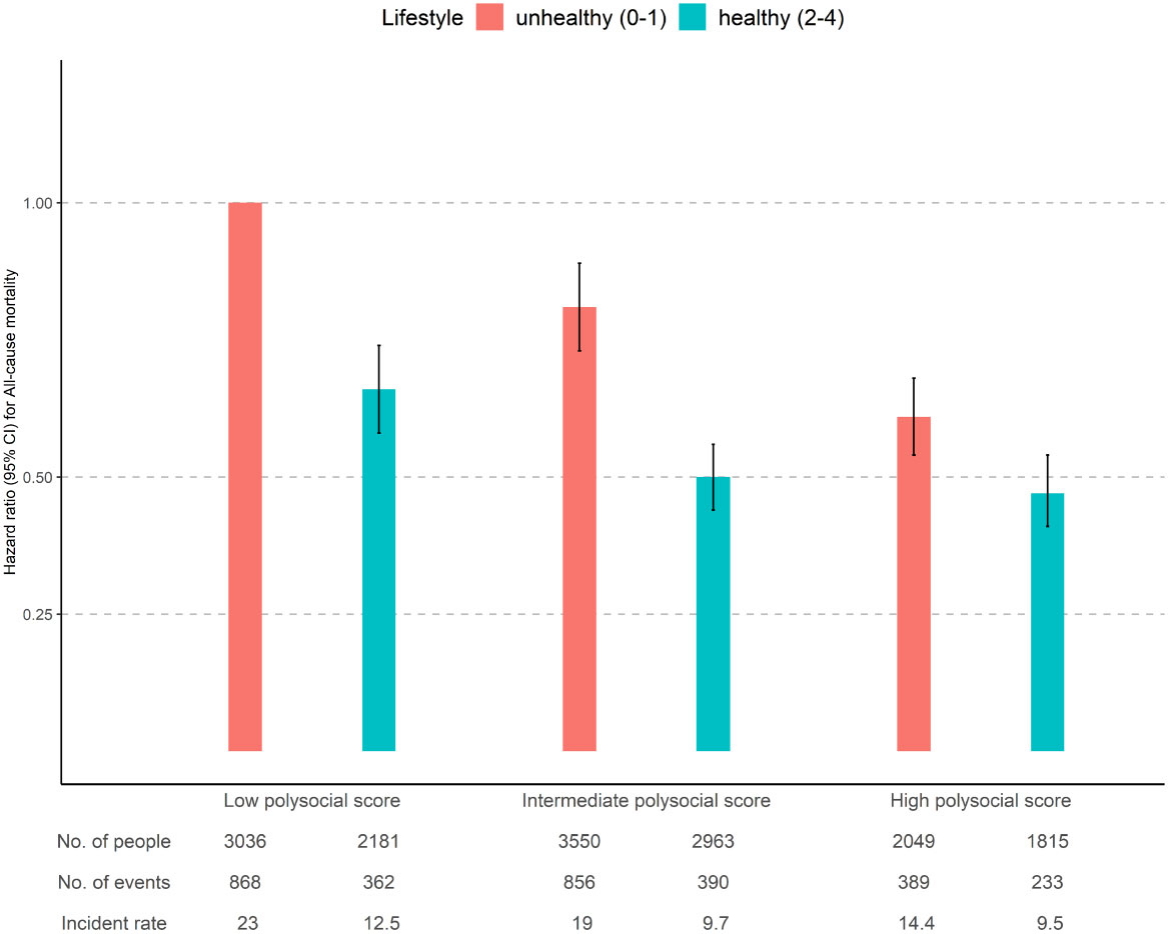
The joint effect of composite lifestyle score and polysocial score on all-cause mortality (*n* = 15 594). Notes: CI, Confidence Interval. Hazard ratios were adjusted for age, sex, and ethnicity. Unhealthy lifestyle = 0 or 1 on the composite healthy lifestyle score, healthy lifestyle = 2,3 and 4 on the composite healthy lifestyle score. The reference group had an unhealthy lifestyle and a low polysocial score

Figure 2 shows the joint effect of lifestyle (healthy and unhealthy) and the polysocial score (high, intermediate and low) on mortality. Among participants with a low polysocial score, those with a healthy lifestyle had a 34% (95% CI = 26–42%) lower hazard of mortality than participants with an unhealthy lifestyle. Compared to those with an unhealthy lifestyle and a low polysocial score, the HR was 0.61 (95% CI = 0.54–0.68) and 0.47 (95% CI = 0.41–0.54) for an unhealthy and healthy lifestyle among those with a high polysocial score. We found that compared with those living in an unfavorable social environment, the beneficial effects of a healthy lifestyle decreased largely among participants living in a favorable social environment. Results did not substantially change in the stratified analyses by age (at least or less than 60 years) or sex (Supplementary figures S10–S13).

## Discussion

In this cohort study of over 15 000 adults with frailty, adherence to a healthy lifestyle, particularly not smoking, adequate physical activity and a healthy diet, was associated with a lower mortality. The favorable association of a healthy lifestyle with mortality was found across all categories of social environment, suggesting that the increased mortality related to living in an undesirable social environment could be offset by a healthy lifestyle. Although no significant interaction effects were observed between lifestyle and social environment, the association between a healthy lifestyle and mortality amplified among frail individuals living in an undesirable social environment. Our findings highlighted the importance of adopting a healthy lifestyle for reducing mortality among people with frailty, particularly those living in an unfavorable social environment.

The reduction of mortality by adhering to a healthy lifestyle has been well-reported among adults.^13^^,^^14^ However, whether the benefits of a healthy lifestyle remain among physically frail individuals is not well understood. The UK Biobank recruited over half a million participants and had a long follow-up, providing an opportunity to answer this research question. We found that adherence to a healthy lifestyle persisted to be associated with a risk reduction in death among people with frailty. We found that frail individuals were behaviorally diverse, and these lifestyle differences were associated with different mortality. Our results suggest that lifestyle changes, which are low-cost and scalable strategies for health promotion, could mitigate frailty-induced health risks and promote resilience among frail individuals. Although many interventions have been proposed for clinical frailty management, the effectiveness of these strategies, particularly those targeting the syndrome per se instead of alleviating symptoms, is not fully supported by empirical evidence.^8^ Findings from a recent multinational clinical trial suggest that a multicomponent intervention comprising moderate-intensity physical activity and nutritional counseling could reduce the incidence of mobility disability among physically frail older adults.^30^ Our results highlighted the importance of promoting a healthy lifestyle among individuals with frailty.

Little is known about whether and how lifestyle interacts with the social environment in shaping health. Some individuals with frailty live in a desirable social environment with a stable economic condition and high social support, while others suffer from unfavorable social conditions. Our study was among the first to explore the joint association of lifestyle and social environment with health among people with frailty. The favorable association of lifestyle with mortality was found across all social environment categories, suggesting that the social environment-induced increase in mortality could be offset to a certain degree by a healthy lifestyle. Although we did not reveal a significant interaction between lifestyle and social environment, the association of a healthy lifestyle with mortality amplified among frail individuals living in an undesirable social environment. Our findings suggest that socially disadvantaged frail individuals might benefit the most from adherence to a healthy lifestyle.

Our study has several strengths. First, previous work focused on the health benefits of adhering to a healthy lifestyle among adults. To our knowledge, this is the first study to examine the association between lifestyle factors and mortality among people with frailty, which lays the foundation for future research in identifying socioeconomic, lifestyle and health features that could mitigate frailty-induced health risks and promote resilience among adults becoming frail. Additionally, in contrast to previous frailty-related research, which included a relatively small sample size of frail participants,^31–33^ we utilized data from a large sample of frail participants from the UK Biobank to perform the stratified and joint analyses. Finally, we used a polysocial score approach to measure social factors from different domains in an aggregated way. This tool has advantages over traditional approaches, which considered social factors separately, and can capture the multidimensional and interactive features of the social environment.

We acknowledge several limitations. First, we measured lifestyle factors and social determinants at baseline. Lifestyle factors and social determinants are dynamic and might change over time. Future research needs to characterize the trajectories of the lifestyle and social factors to better understand dynamics in the joint effect of lifestyles and social environment on mortality. Second, 87.1% of our analytic sample was White. Future research is needed to examine the association of lifestyles and social environment with mortality among other racial and ethnic groups. Third, in the absence of a universally accepted operational definition, numerous frailty assessment tools have been proposed over the past two decades, each rooted in distinct theories, designed for a specific objectives, and applied in different settings.^34^^,^^35^ Considerable heterogeneity exists in the theoretical foundations guiding the development of frailty assessment tools, and the level of consensus among these instruments varies substantially.^36^ In the present study, we conceptualized frailty as a distinct medical syndrome with specific biological basis and pathogenesis and, therefore, used the theory-guided and well-validated PFP approach for its assessment. Nevertheless, the concept of frailty could be understood from a more integrative perspective incorporating psychological and social aspects.^37^ Future research needs to understand the dynamic relationship between frailty, measured by a multidimensional instrument (e.g. Tilburg Frailty Indicator),^38^ and changes in physical, psychological and social functioning. Fourth, we dichotomized each social variable to create the polysocial score, which might lead to the loss of useful information contained in the original continuous variables. We transformed continuous variables into binary variables mainly to provide easy interpretation and presentation of results. A comprehensive evaluation of the psychometric properties of the polysocial score constructed by continuous variables might be needed to better understand the differences between the two approaches. Fifth, we adopted a mixture of objective measurements (e.g. grip strength) and self-reported items (e.g. weight loss) to measure frailty. Although evidence suggests self-reported alternatives are usually reliable,^39^^,^^40^ they are inevitably subject to recall bias, potentially leading to misclassification. Sixth, the present study did not consider the changes in lifestyle factors over time; subsequent analyses could explore the impact of lifestyle changes on health outcomes. Finally, we did not include social factors in the healthcare domain (long-term insurance coverage, etc.) in constructing the polysocial score due to data unavailability in the UK Biobank, which has been identified as an important component of the polysocial score for mortality.^22^ Future research needs to examine whether healthcare factors are still a crucial part of a polysocial score among people with frailty.

In conclusion, we found that adherence to a healthy lifestyle, particularly not smoking, adequate physical activity and a healthy diet, was associated with a lower mortality among frail adults. The health benefits of adopting a healthy lifestyle were universal across social environment categories, especially among those living in an undesirable social environment. Lifestyle interventions might help reduce mortality and prolong survival across entire frail populations, even in socially disadvantaged groups.

## Supplementary Material

ckae003_Supplementary_Data

## Data Availability

Data used in the current study were from the UK Biobank study with application number 51450. Researchers can request the data we used upon approval from the UK Biobank study. Key points Adherence to a healthy lifestyle, particularly not smoking, adequate physical activity and a healthy diet, was associated with a lower mortality among frail adults. The health benefits of adopting a healthy lifestyle were universal across social environment categories. Among frail adults living in an undesirable social environment, adopting a healthy lifestyle could prolong their survival.
