# Supplementary material for: Associations between healthy lifestyle and mortality across different social environments: a study among adults with frailty from the UK Biobank
Source: Eur J Public Health. 2024 Jan 30;34(2):218–24. doi: 10.1093/eurpub/ckae003 (PMC10990525; doi:10.1093/eurpub/ckae003)
Supplement: ckae003_Supplementary_Data [file ckae003_supplementary_data.docx]

**SUPPLEMENTARY MATERIALS**

- **Supplementary Table 1.** Operational definition of each of five criteria for creating the frailty phenotype score in the UK Biobank.
- **Supplementary Table 2.** Description of lifestyle factors.
- **Supplementary Table 3.** Definition of a favorable and reference level for social factors.
- **Supplementary Table 4.** Association between lifestyle factors and all-cause mortality by age (at least or less than 60 years).
- **Supplementary Table 5.** Association between lifestyle factors and all-cause mortality by sex.
- **Supplementary Figure 1.** Flow chart of sample selection.
- **Supplementary Figure 2.** Distribution of the polysocial score for all-cause mortality (n=15,594).
- **Supplementary Figure 3.** Association of lifestyle factors with all-cause mortality across polysocial score categories for participants who were frail and aged at least 60 years at baseline (n=7,519).
- **Supplementary Figure 4.** Association of lifestyle factors with all-cause mortality across polysocial score categories for participants who were frail and aged less than 60 years at baseline (n=8,075).
- **Supplementary Figure 5.** Association of lifestyle factors with all-cause mortality across polysocial score categories for participants who were frail and male (n=5,822).
- **Supplementary Figure 6.** Association of lifestyle factors with all-cause mortality across polysocial score categories for participants who were frail and female (n=9,722).
- **Supplementary Figure 7.** Survival plot for healthy lifestyle score, classified by polysocial score (high, intermediate, and low).
- **Supplementary Figure 8.** K-M plot for healthy lifestyle score among participants who were frail and aged at least 60 years at baseline, classified by polysocial score (n=7,519).
- **Supplementary Figure 9.** Sensitivity analysis- K-M plot for healthy lifestyle score, classified by polysocial score (n=15, 594).
- **Supplementary Figure 10.** The joint effect of composite lifestyle score and polysocial score on all-cause mortality for participants who were frail and aged at least 60 years at baseline (n=7,519).
- **Supplementary Figure 11.** The joint effect of composite lifestyle score and polysocial score on all-cause mortality for participants who were frail and aged less than 60 years at baseline (n=8,075).
- **Supplementary Figure 12.** The joint effect of composite lifestyle score and polysocial score on all-cause mortality for participants who were frail and male (n=5,822).
- **Supplementary Figure 13.** The joint effect of composite lifestyle score and polysocial score on all-cause mortality for participants who were frail and female (n=9,722).

**Supplementary Table 1.** Operational definition of each of five criteria for creating the frailty phenotype score in the UK Biobank.

| **Criterion** | **Operational definition in the UK Biobank** |
| --- | --- |
| Weakness | Measured grip strength (sex and body-mass index adjusted cutoffs taken from Fried and colleagues) ^a^ |
| Slowness | Self-reported: “How would you describe your usual walking pace?” (response: slow=1, other=0) ^b^ |
| Exhaustion | Self-reported: “Over the past two weeks, how often have you felt tired or had little energy?” (response: more than half the days or nearly every day=1, other=0) ^b^ |
| Physical inactivity | Self-reported: UK Biobank physical activity questionnaire. We classified the responses into: none (no physical activity in the last 4 weeks), low (light DIY activity (e.g., pruning, watering the lawn) only in the past 4 weeks), medium (heavy DIY activity (e.g., weeding, lawn mowing, carpentry and digging), walking for pleasure, or other exercises in the past 4 weeks), and high (strenuous sports in the past 4 weeks) (response: none or light activity with a frequency of once per week or less=1, medium or heavy activity, or light activity more than once per week=0) ^c^ |
| Shrinking | Self-reported: “Compared with one year ago, has your weight changed?” (response: yes, lost weight=1, other=0) ^b^ |

Note: The slowness criterion was met if the participants answered “Slow” to the question, “How would you describe your usual walking pace?”. The weakness criterion was met when handgrip strength was less than or equal to the sex- and body mass index-specific cut-points (worst quintiles). The exhaustion criterion was met if the participants answered, “Nearly every day” or “More than half the days” when asked, “Over the past two weeks, how often have you felt tired or had little energy?”. The inactivity criterion was met if the participants’ responses were classified as none or light activity with a frequency of once per week or less. The shrinking criterion was met if the participants answered “Yes, lost weight” when asked, “Compared with one year ago, has your weight changed?”. Frailty level was identified by the number of criteria met. Individuals with none were considered “robust/nonfrail”; those meeting one or two criteria were considered “prefrail”; and those with three to five criteria were defined as “frail”.

^a^ Definition used in the original description by Fried and colleagues.

^b^ Approximation based on available variables in UK Biobank assessment center data.

^c^ Definition used in the SHARE adaptation of the frailty phenotype.

**Supplementary Table 2.** Description of lifestyle factors.

| **Lifestyle factors** | **Description** |
| --- | --- |
|  |  |
| **Smoking** | Never smoking or smoking fewer than 100 cigarettes in life was categorized as a healthy level of smoking; current smoking, ever frequent smoking, or smoking more than 100 cigarettes in life was classified as an unhealthy level. |
| **Alcohol consumption** | Daily consumption of 8 grams or fewer for female and 16 grams or fewer for male was categorized as a healthy level; daily consumption of more than 8 grams for female and more than 16 grams for male was classified as an unhealthy level. |
|  |  |
| **Physical activity** | The frequency (number of times in the past 4 weeks), duration (time length of each time), and intensity (using the standardized metabolic equivalent of task [MET]) of four activities: walking for pleasure, heavy DIY (do-it-yourself, e.g., weeding, carpentry, digging), strenuous sports, and other exercises (e.g., bowling, swimming, cycling). The MET value for walking for pleasure, heavy DIY, strenuous sports, and other exercises is 3.5, 5.5, 8.0, and 4.0, respectively. For physical activity, we calculated a weekly metabolic equivalent time period for each activity by multiplying the frequency, duration, and the MET value, and computed the total weekly metabolic equivalent time period by summing the time spent on each activity. We classified participants into three tertiles according to the total weekly metabolic equivalent time period. The top layer was categorized as a healthy level; the other two layers was classified as an unhealthy level. |
|  |  |
| **Diet** | The diet recommendation included adequate intake of whole grains, vegetables, vegetable oils, shellfish, fruit, and dairy products and reduced intake of unprocessed meats, processed meats, refined grains, and sugar-sweetened beverages. Satisfying no less than five items of the food recommendation was categorized as a healthy level; satisfying less than or equal to four items of the food recommendation was categorized as an unhealthy level. |

**Supplementary Table 3.** Definition of a favorable and reference level for social factors.

| **Social factors** | **Definition of a favorable level** | **Definition of a reference level** |
| --- | --- | --- |
| **Socioeconomic status:** |  |  |
| **Highest education level** | The participants’ highest education level was college or above. | The participants’ highest education level was lower than college. |
|  |  |  |
| **Education score** | The participants’ education score was above the median of education score. | The participants’ education score was below the median of education score. |
|  |  |  |
| **Employment status** | The participants’ employment status was in paid employment or self-employed. | The participants neither had paid employment nor were self-employed. |
|  |  |  |
| **Psychosocial factors:** |  |  |
| **Living situation** | The participants lived with household members. | The participants lived alone . |
|  |  |  |
| **Social support** | The participants were able to confide in anyone close to them at least once a week. | The participants were unable to confide in anyone close to them at least once a week. |
|  |  |  |
| **Social activity** | The participants attended any group activities at least once a week. | The participants attended any group activities less than once a week. |
|  |  |  |
| **Social isolation** | The participants visited their friends/family or had them to visit more than once a week. | The participants visited their friends/family or had them to visit once a week or less. |
|  |  |  |
| **Emotional distress** | The participants had no experience of illness, injury, bereavement, or stress. | The participants experienced illness, injury, bereavement, or stress. |
|  |  |  |
| **Psychiatric disorder** | The had no or rare feeling of loneliness. | The participants had a usual feeling of loneliness. |
|  |  |  |
| **Neighborhood and living environment:** |  |  |
| **Townsend deprivation index** | The participants’ Townsend deprivation index was below the median. | The participants’ Townsend deprivation index was above the median. |
|  |  |  |
| **Crime rate** | The crime score of the participants neighborhood was below the median. | The crime score of the participants neighborhood was above the median. |
|  |  |  |
| **Housing quality** | The participants’ housing score was below the median. | The participants’ housing score was above the median. |
|  |  |  |
| **Accommodation ownership** | The participants owned their current accommodation outright. | The participants did not own their current accommodation. |
|  |  |  |
| **Type of house** | The participants lived in a house. | The participants lived in an apartment. |
|  |  |  |
| **Greenspace** | Within the areas of the participants’ home location buffer, the percentages of land classified as greenspace was above the median. | Within the areas of the participants’ home location buffer, the percentages of land classified as greenspace was below the median. |
|  |  |  |
| **Bluespace** | Within the areas of the participants’ home location buffer, the percentages of land classified as bluespace was above the median. | Within the areas of the participants’ home location buffer, the percentages of land classified as bluespace was below the median. |
|  |  |  |
| **Natural environment** | Within the areas of the participants’ home location buffer, the percentages of land classified as natural land was above the median. | Within the areas of the participants’ home location buffer, the percentages of land classified as natural land was below the median. |

**Supplementary Table 4.** Association between lifestyle factors and all-cause mortality by age (at least or less than 60 years).

|  | At least 60 years | | | Less than 60 years | | |
| --- | --- | --- | --- | --- | --- | --- |
| Lifestyle factors | Mortality rate per 1,000 PYs | HR (95% CI) | | Mortality per 1,000 PYs | HR (95% CI) | |
|  |  | Unadjusted | Adjusted ^a^ |  | Unadjusted | Adjusted ^a^ |
| **Smoking** |  |  |  |  |  |  |
| Unhealthy | 29.1 | Ref. | Ref. | 12.1 | Ref. | Ref. |
| Healthy | 15.3 | 0.52 (0.48-0.57) | 0.62 (0.56-0.68) | 5.6 | 0.46 (0.41-0.53) | 0.56 (0.48-0.64) |
|  |  |  |  |  |  |  |
| **Alcohol consumption** |  |  |  |  |  |  |
| Unhealthy | 25.4 | Ref. | Ref. | 10.3 | Ref. | Ref. |
| Healthy | 21.8 | 0.86 (0.78-0.94) | 1.00 (0.92-1.10) | 8.2 | 0.80 (0.70-0.92) | 0.97 (0.84-1.11) |
|  |  |  |  |  |  |  |
| **Physical activity** |  |  |  |  |  |  |
| Unhealthy | 23.3 | Ref. | Ref. | 9.0 | Ref. | Ref. |
| Healthy | 17.6 | 0.75 (0.63-0.91) | 0.70 (0.58-0.84) | 5.7 | 0.64 (0.45-0.90) | 0.61 (0.43-0.86) |
|  |  |  |  |  |  |  |
| **Diet** |  |  |  |  |  |  |
| Unhealthy | 23.3 | Ref. | Ref. | 9.0 | Ref. | Ref. |
| Healthy | 20.0 | 0.86 (0.75-0.97) | 0.88 (0.78-1.00) | 6.9 | 0.76 (0.61-0.95) | 0.80 (0.64-1.00) |
|  |  |  |  |  |  |  |
| **Healthy lifestyle** |  |  |  |  |  |  |
| Continuous |  | 0.75 (0.71-0.78) | 0.82 (0.78-0.87) |  | 0.67 (0.62-0.73) | 0.76 (0.70-0.82) |
| Categorical |  |  |  |  |  |  |
| 0 - 1 | 28.4 | Ref. | Ref. | 11.2 | Ref. | Ref. |
| 2 - 4 | 16.1 | 0.56 (0.51-0.61) | 0.66 (0.60-0.72) | 6.0 | 0.53 (0.47-0.61) | 0.65 (0.56-0.74) |

Abbreviation: PYs, person-years; HR, Hazard Ratio; CI, Confidence Interval.

^a^ Adjusted for sex and ethnicity (Whites & Others).

| **Supplementary Table 5.** Association between lifestyle factors and all-cause mortality by sex.   \|  \| Male \| \| \| Female \| \| \| \| --- \| --- \| --- \| --- \| --- \| --- \| --- \| \| Lifestyle factors \| Mortality rate per 1,000 PYs \| HR (95% CI) \| \| Mortality per 1,000 PYs \| HR (95% CI) \| \| \| Unadjusted \| Adjusted ^a^ \| Unadjusted \| Adjusted ^a^ \| \| **Smoking** \|  \|  \|  \|  \|  \|  \| \| Unhealthy \| 27.9 \| Ref. \| Ref. \| 14.4 \| Ref. \| Ref. \| \| Healthy \| 15.3 \| 0.54 (0.49-0.61) \| 0.62 (0.55-0.70) \| 7.9 \| 0.55 (0.49-0.61) \| 0.58 (0.52-0.65) \| \|  \|  \|  \|  \|  \|  \|  \| \| **Alcohol consumption** \|  \|  \|  \|  \|  \|  \| \| Unhealthy \| 25.3 \| Ref. \| Ref. \| 11.0 \| Ref. \| Ref. \| \| Healthy \| 22.4 \| 0.88 (0.80-0.97) \| 0.98 (0.88-1.08) \| 10.8 \| 0.98 (0.87-1.11) \| 1.02 (0.90-1.15) \| \|  \|  \|  \|  \|  \|  \|  \| \| **Physical activity** \|  \|  \|  \|  \|  \|  \| \| Unhealthy \| 23.9 \| Ref. \| Ref. \| 11.0 \| Ref. \| Ref. \| \| Healthy \| 17.0 \| 0.71 (0.57-0.87) \| 0.65 (0.53-0.81) \| 8.3 \| 0.75 (0.58-0.97) \| 0.71 (0.55-0.92) \| \|  \|  \|  \|  \|  \|  \|  \| \| **Diet** \|  \|  \|  \|  \|  \|  \| \| Unhealthy \| 23.6 \| Ref. \| Ref. \| 11.0 \| Ref. \| Ref. \| \| Healthy \| 21.8 \| 0.92 (0.79-1.07) \| 0.85 (0.73-1.00) \| 9.9 \| 0.90 (0.76-1.05) \| 0.86 (0.74-1.02) \| \|  \|  \|  \|  \|  \|  \|  \| \| **Healthy lifestyle** \|  \|  \|  \|  \|  \|  \| \| Continuous \|  \| 0.77 (0.73-0.82) \| 0.82 (0.77-0.87) \|  \| 0.77 (0.73-0.82) \| 0.79 (0.74-0.84) \| \| Categorical \|  \|  \|  \|  \|  \|  \| \| 0 - 1 \| 27.3 \| Ref. \| Ref. \| 13.4 \| Ref. \| Ref. \| \| 2 - 4 \| 16.2 \| 0.59 (0.53-0.66) \| 0.65 (0.58-0.73) \| 8.4 \| 0.63 (0.56-0.70) \| 0.65 (0.59-0.73) \|   Abbreviation: PYs, person-years; HR, Hazard Ratio; CI, Confidence Interval.  ^a^ Adjusted for age and ethnicity (Whites & Others). |
| --- | --- | --- | --- | --- | --- | --- | --- | --- | --- | --- | --- | --- | --- | --- | --- | --- | --- | --- | --- | --- | --- | --- | --- | --- | --- | --- | --- | --- | --- | --- | --- | --- | --- | --- | --- | --- | --- | --- | --- | --- | --- | --- | --- | --- | --- | --- | --- | --- | --- | --- | --- | --- | --- | --- | --- | --- | --- | --- | --- | --- | --- | --- | --- | --- | --- | --- | --- | --- | --- | --- | --- | --- | --- | --- | --- | --- | --- | --- | --- | --- | --- | --- | --- | --- | --- | --- | --- | --- | --- | --- | --- | --- | --- | --- | --- | --- | --- | --- | --- | --- | --- | --- | --- | --- | --- | --- | --- | --- | --- | --- | --- | --- | --- | --- | --- | --- | --- | --- | --- | --- | --- | --- | --- | --- | --- | --- | --- | --- | --- | --- | --- | --- | --- | --- | --- | --- | --- | --- | --- | --- | --- | --- | --- | --- | --- | --- | --- | --- | --- | --- | --- | --- | --- | --- | --- | --- | --- | --- | --- | --- | --- | --- | --- | --- | --- |

**Supplementary Figure 1.** Flow chart of sample selection.


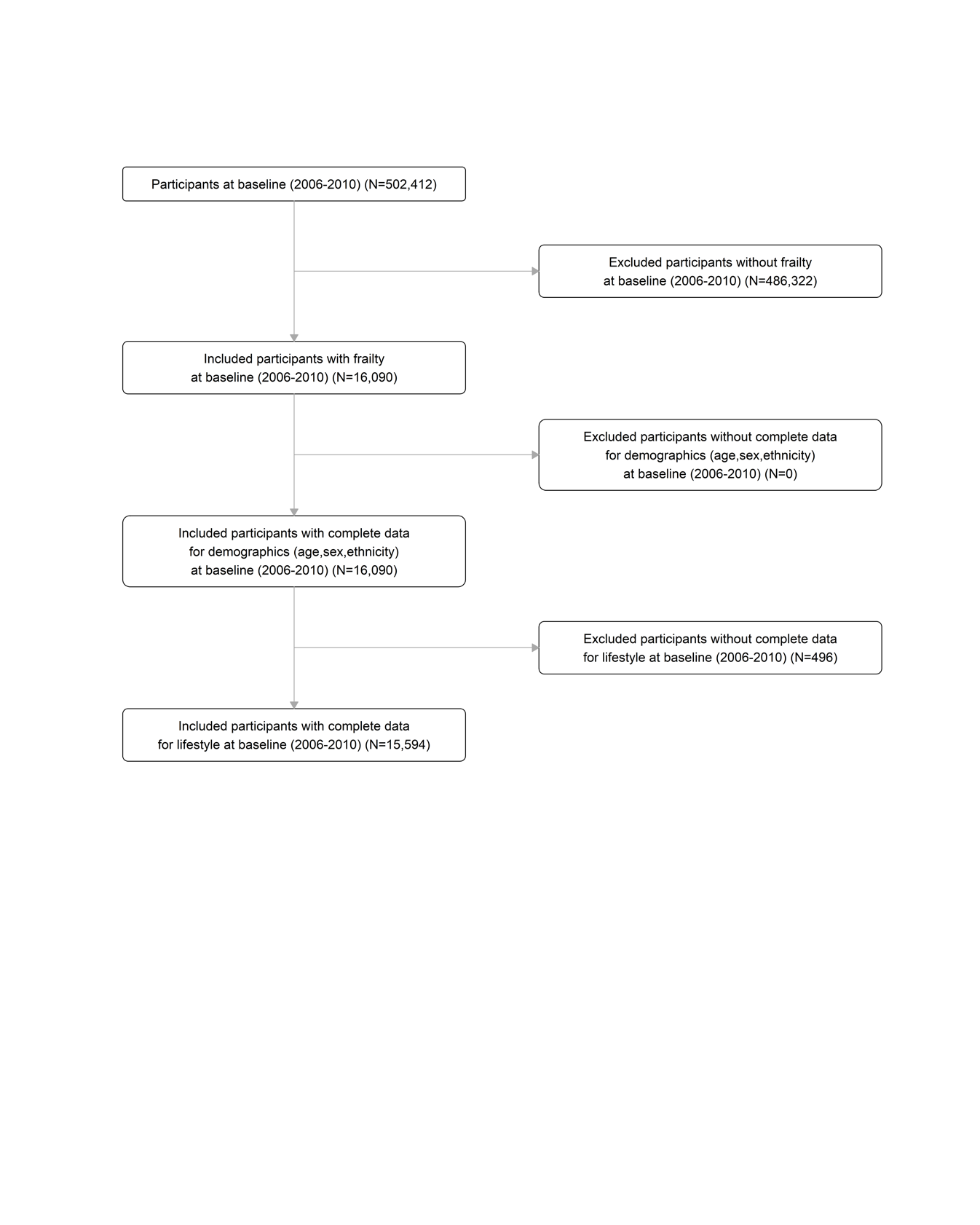


**Supplementary Figure 2.** Distribution of the polysocial score for all-cause mortality (n=15,594).


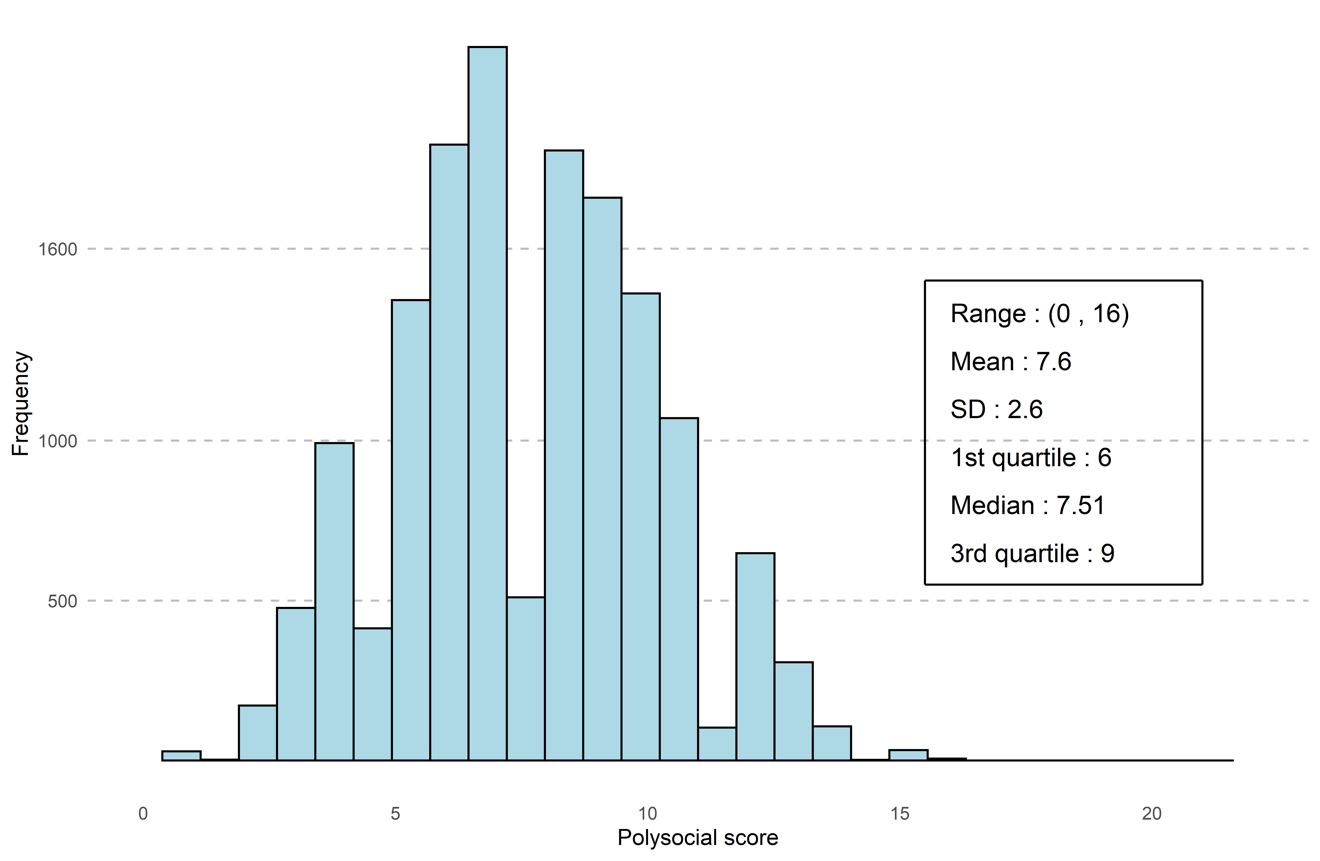


**Supplementary Figure 3.** Association of lifestyle factors with all-cause mortality across polysocial score categories for participants who were frail and aged at least 60 years at baseline (n=7,519).


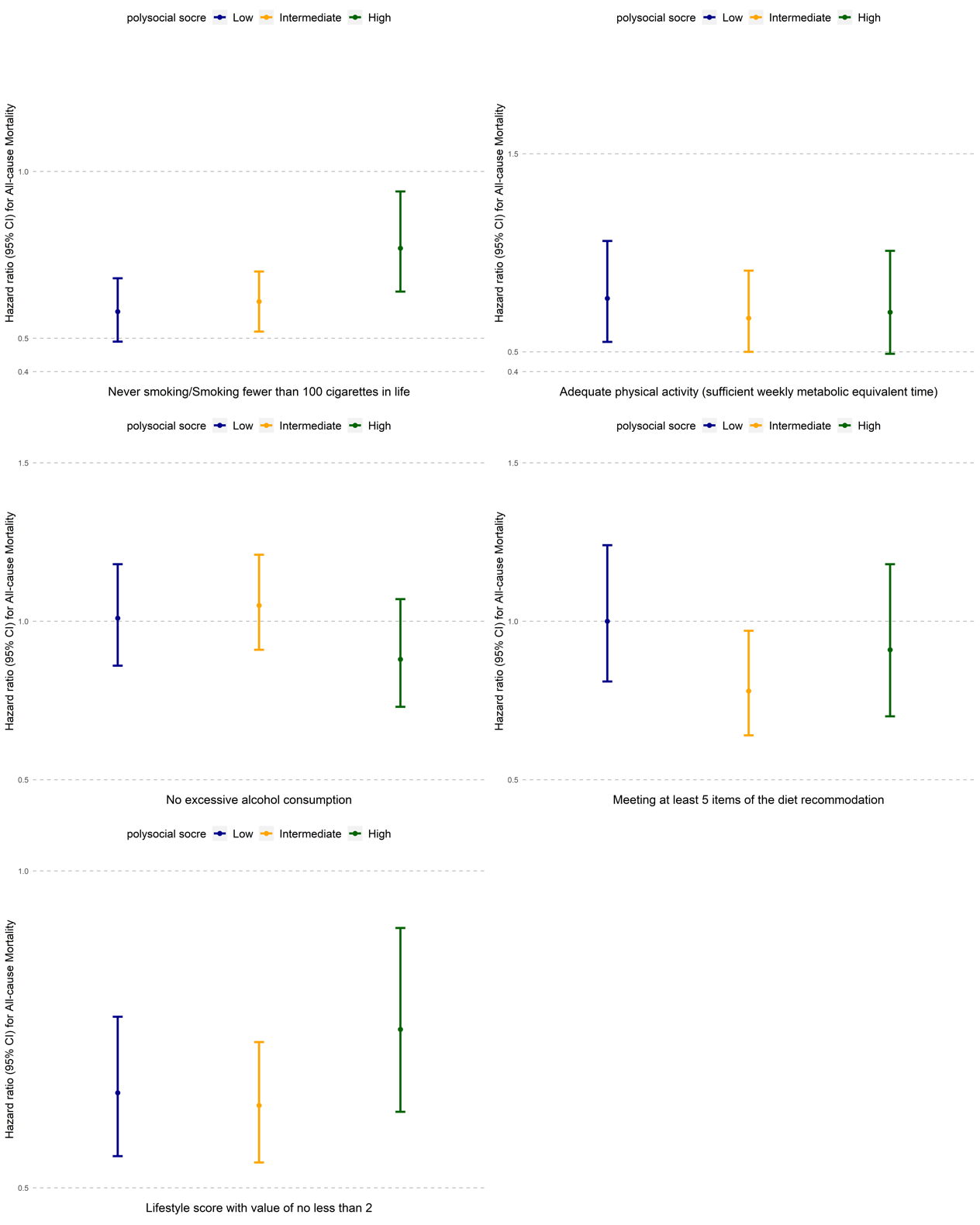


Note: CI, Confidence Interval. Hazard ratios were adjusted for sex and ethnicity (Whites & Others). Multiplicative interaction was assessed using hazard ratio for the product interaction between each lifestyle factor (smoking, alcohol consumption, physical activity, diet, and the composite healthy lifestyle score) and the continuous polysocial score. For the composite healthy lifestyle score, the unhealthy level represented 0 and 1, and the healthy level represented 2, 3, and 4.

**Supplementary Figure 4.** Association of lifestyle factors with all-cause mortality across polysocial score categories for participants who were frail and aged less than 60 years at baseline (n=8,075).


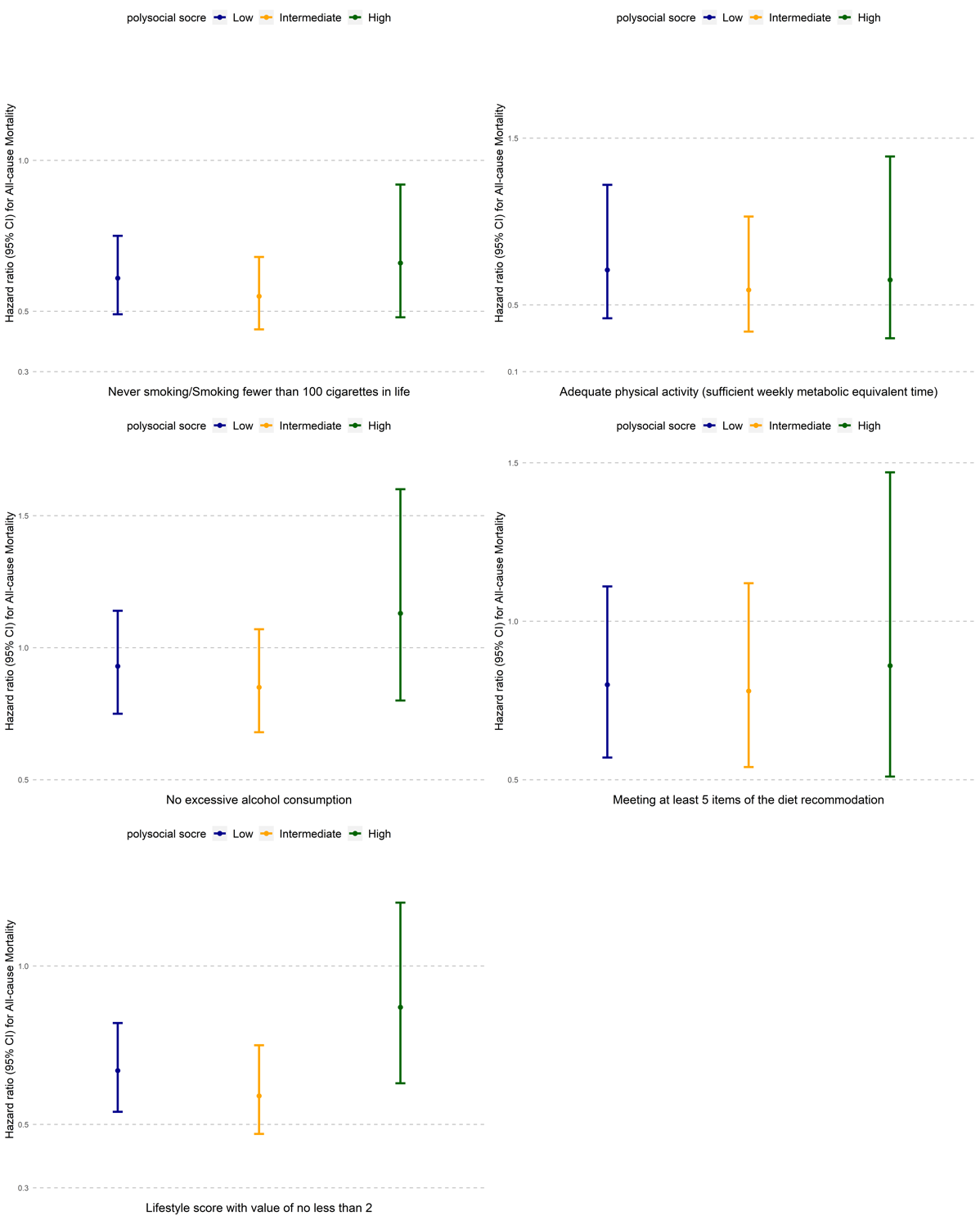


Note: CI, Confidence Interval. Hazard ratios were adjusted for sex and ethnicity (Whites & Others). Multiplicative interaction was assessed using hazard ratio for the product interaction between each lifestyle factor (smoking, alcohol consumption, physical activity, diet, and the composite healthy lifestyle score) and the continuous polysocial score. For the composite healthy lifestyle score, the unhealthy level represented 0 and 1, and the healthy level represented 2, 3, and 4.

**Supplementary Figure 5.** Association of lifestyle factors with all-cause mortality across polysocial score categories for participants who were frail and male (n=5,822).


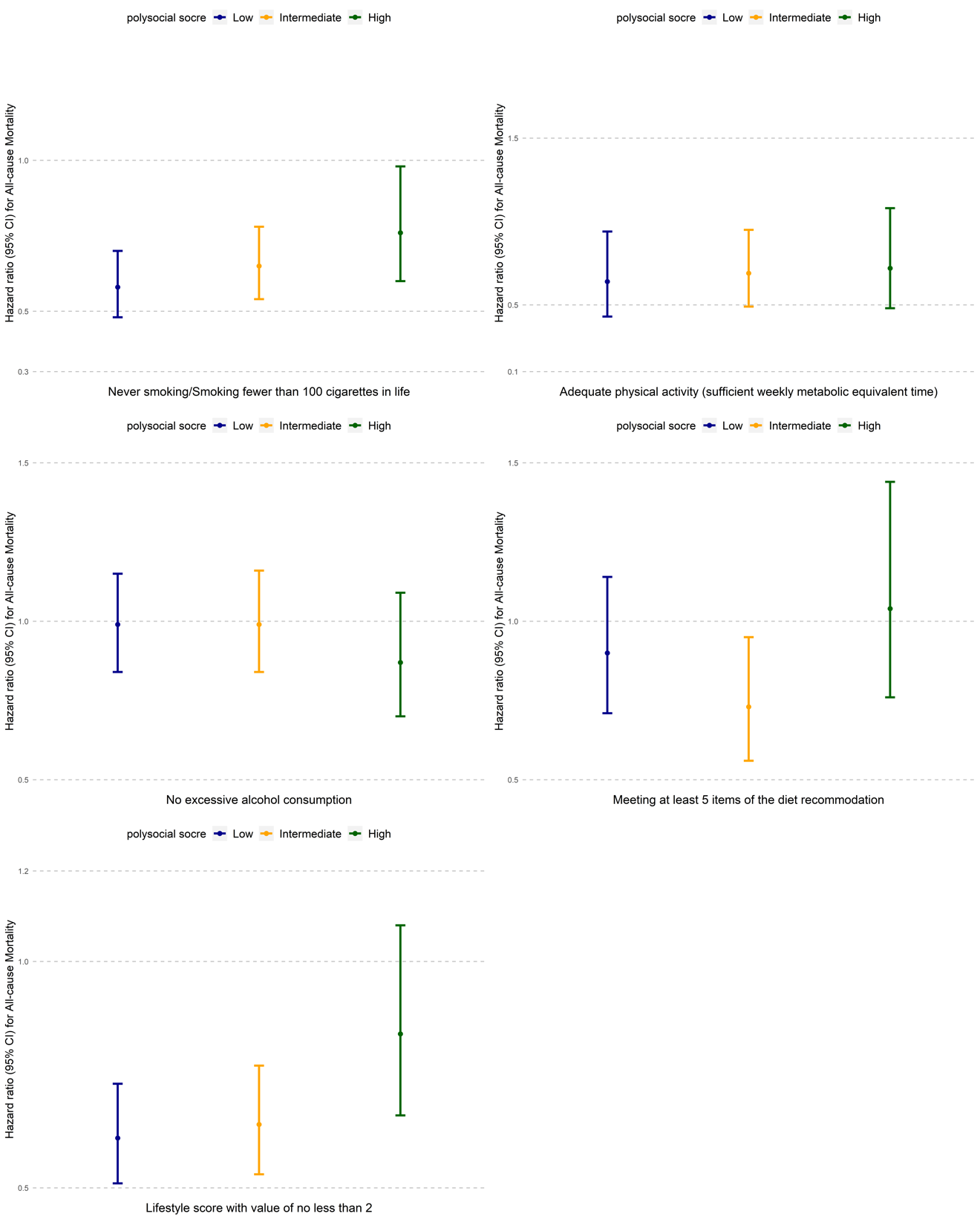


Note: CI, Confidence Interval. Hazard ratios were adjusted for age and ethnicity (Whites & Others). Multiplicative interaction was assessed using hazard ratio for the product interaction between each lifestyle factor (smoking, alcohol consumption, physical activity, diet, and the composite healthy lifestyle score) and the continuous polysocial score. For the composite healthy lifestyle score, the unhealthy level represented 0 and 1, and the healthy level represented 2, 3, and 4.

**Supplementary Figure 6.** Association of lifestyle factors with all-cause mortality across polysocial score categories for participants who were frail and female (n=9,722).


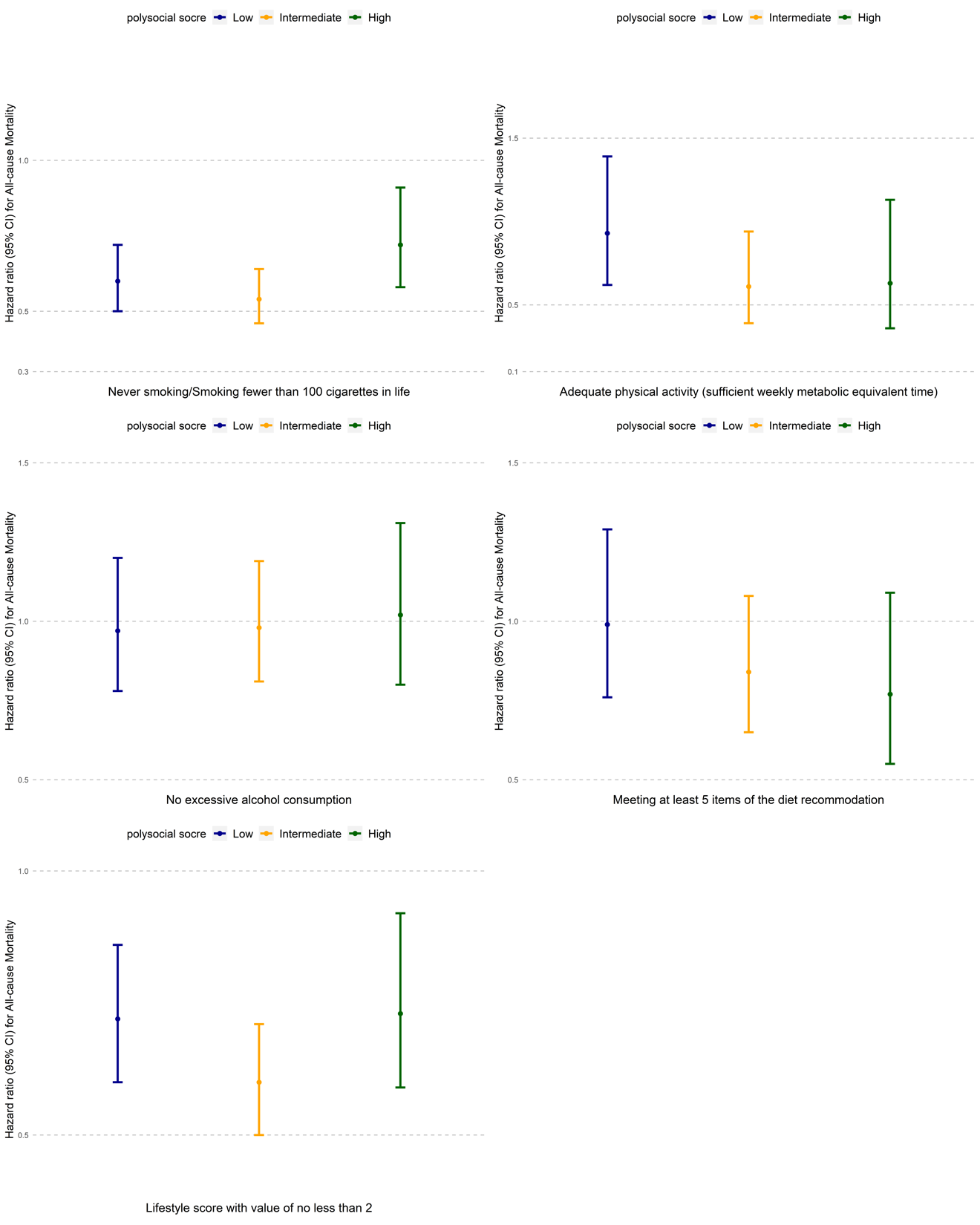


Note: CI, Confidence Interval. Hazard ratios were adjusted for age and ethnicity (Whites & Others). Multiplicative interaction was assessed using hazard ratio for the product interaction between each lifestyle factor (smoking, alcohol consumption, physical activity, diet, and the composite healthy lifestyle score) and the continuous polysocial score. For the composite healthy lifestyle score, the unhealthy level represented 0 and 1, and the healthy level represented 2, 3, and 4.

**Supplementary Figure 7.** Survival plot for healthy lifestyle score, classified by polysocial score (high, intermediate, and low).


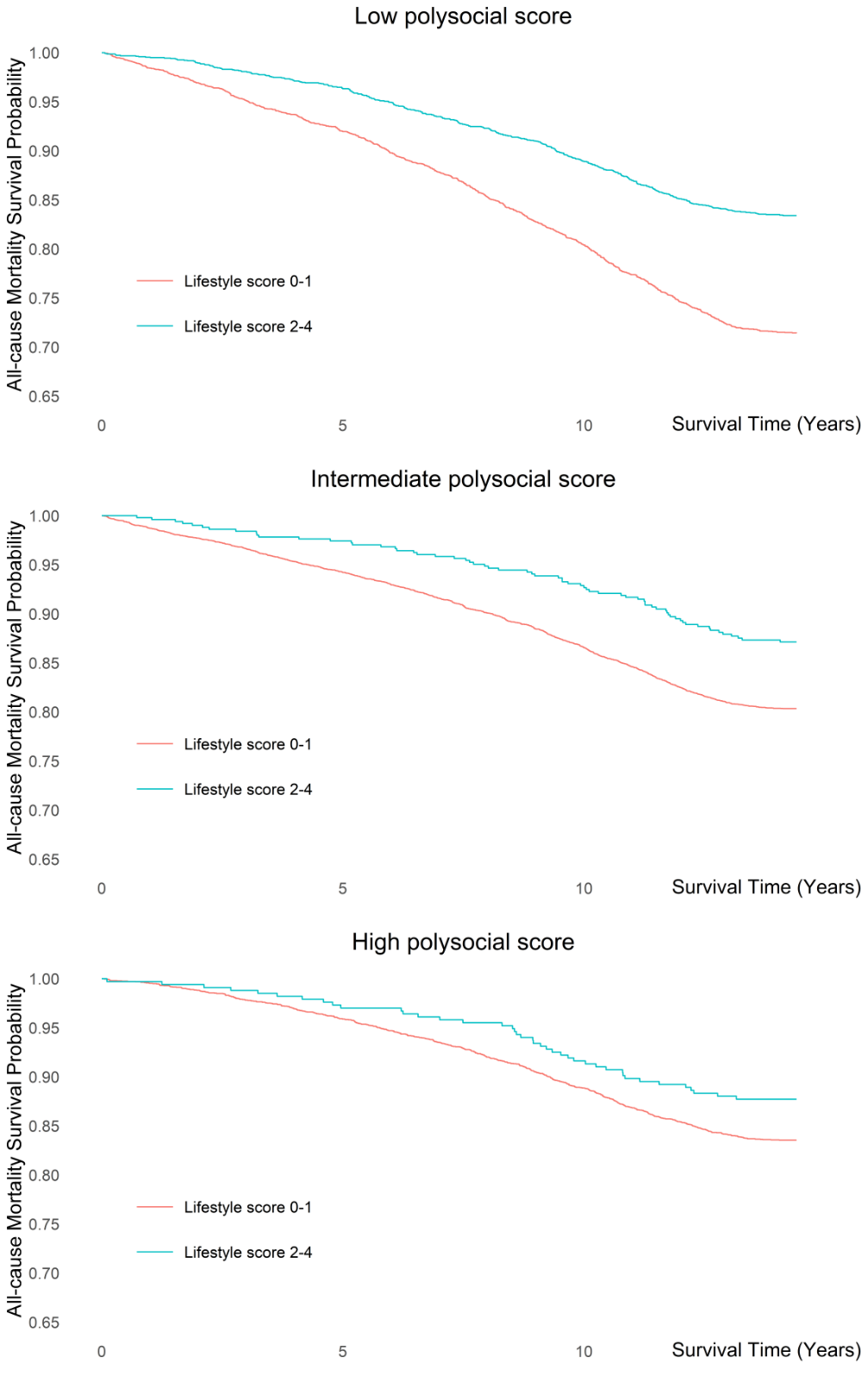


**Supplementary Figure 8.** K-M plot for healthy lifestyle score among participants who were frail and aged at least 60 years at baseline, classified by polysocial score (n=7,519).

**
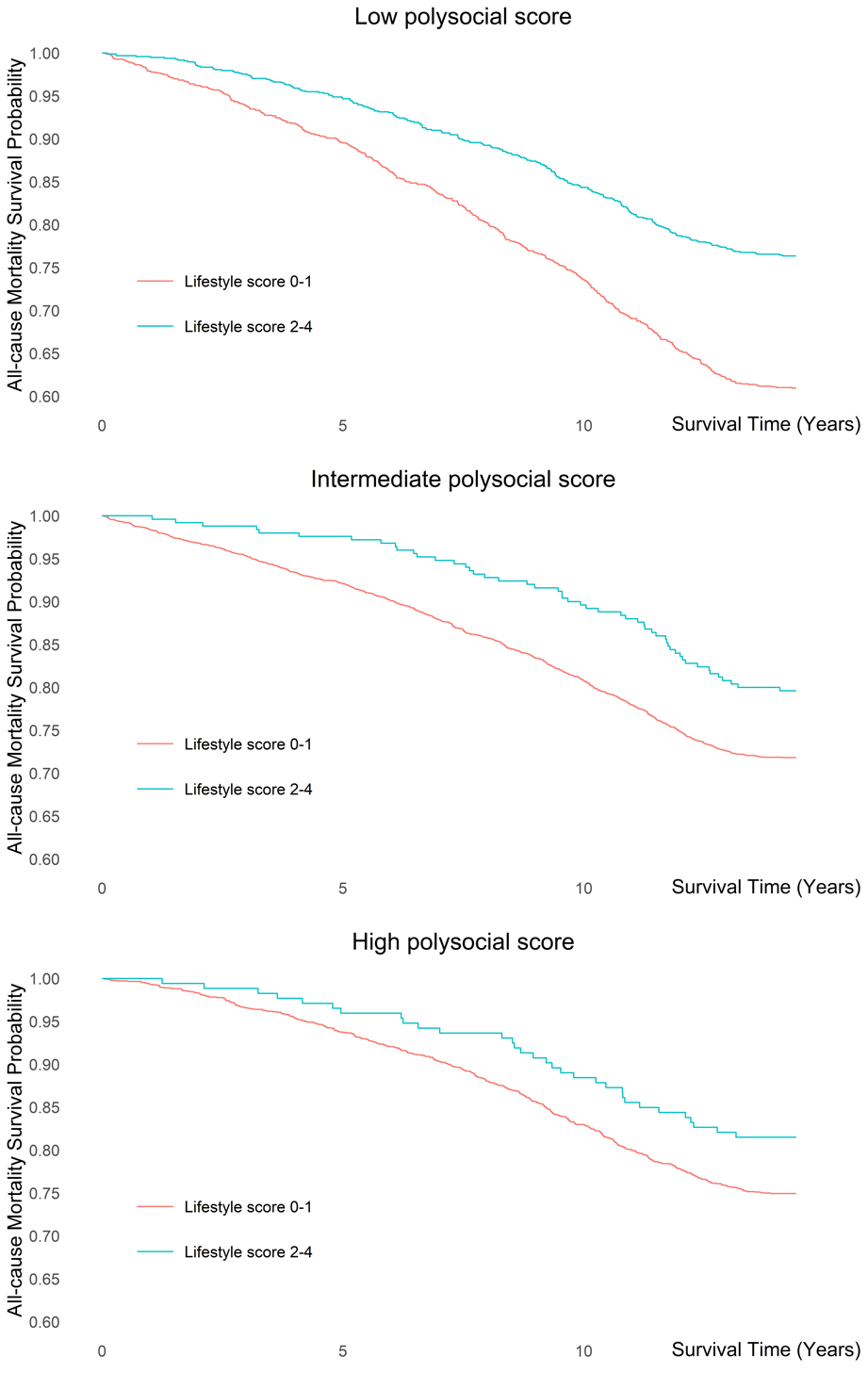
**

**Supplementary Figure 9.** Sensitivity analysis: K-M plot for healthy lifestyle score, classified by polysocial score (n=15, 594).


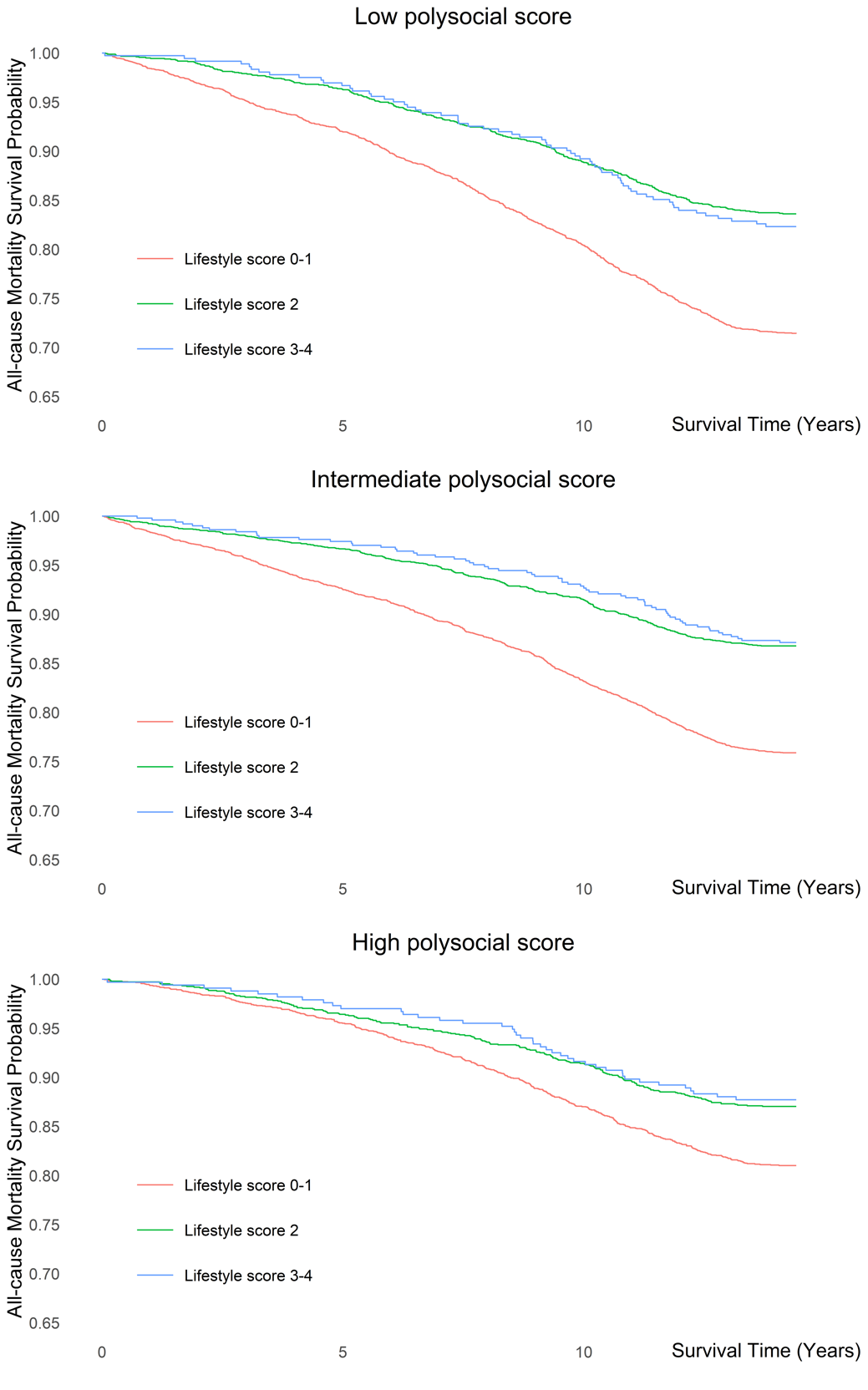


**Supplementary Figure 10.** The joint effect of composite lifestyle score and polysocial score on all-cause mortality for participants who were frail and aged at least 60 years at baseline (n=7,519).


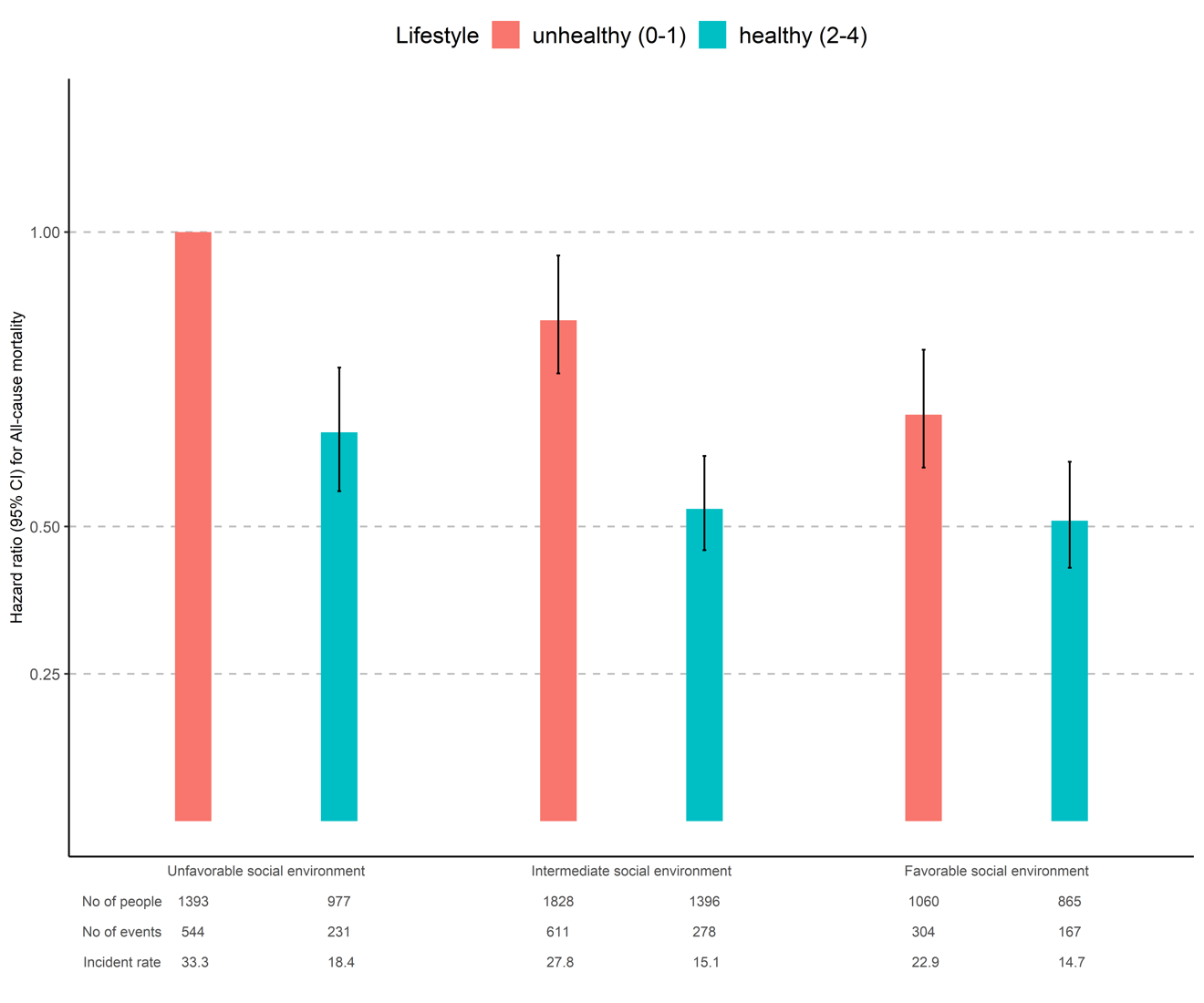


Note: CI, Confidence Interval. Hazard ratios were adjusted for sex and ethnicity (Whites & Others). For the composite healthy lifestyle score, the unhealthy level represented 0 and 1, and the healthy level represented 2,3, and 4.

**Supplementary Figure 11.** The joint effect of composite lifestyle score and polysocial score on all-cause mortality for participants who were frail and aged less than 60 years at baseline (n=8,075).


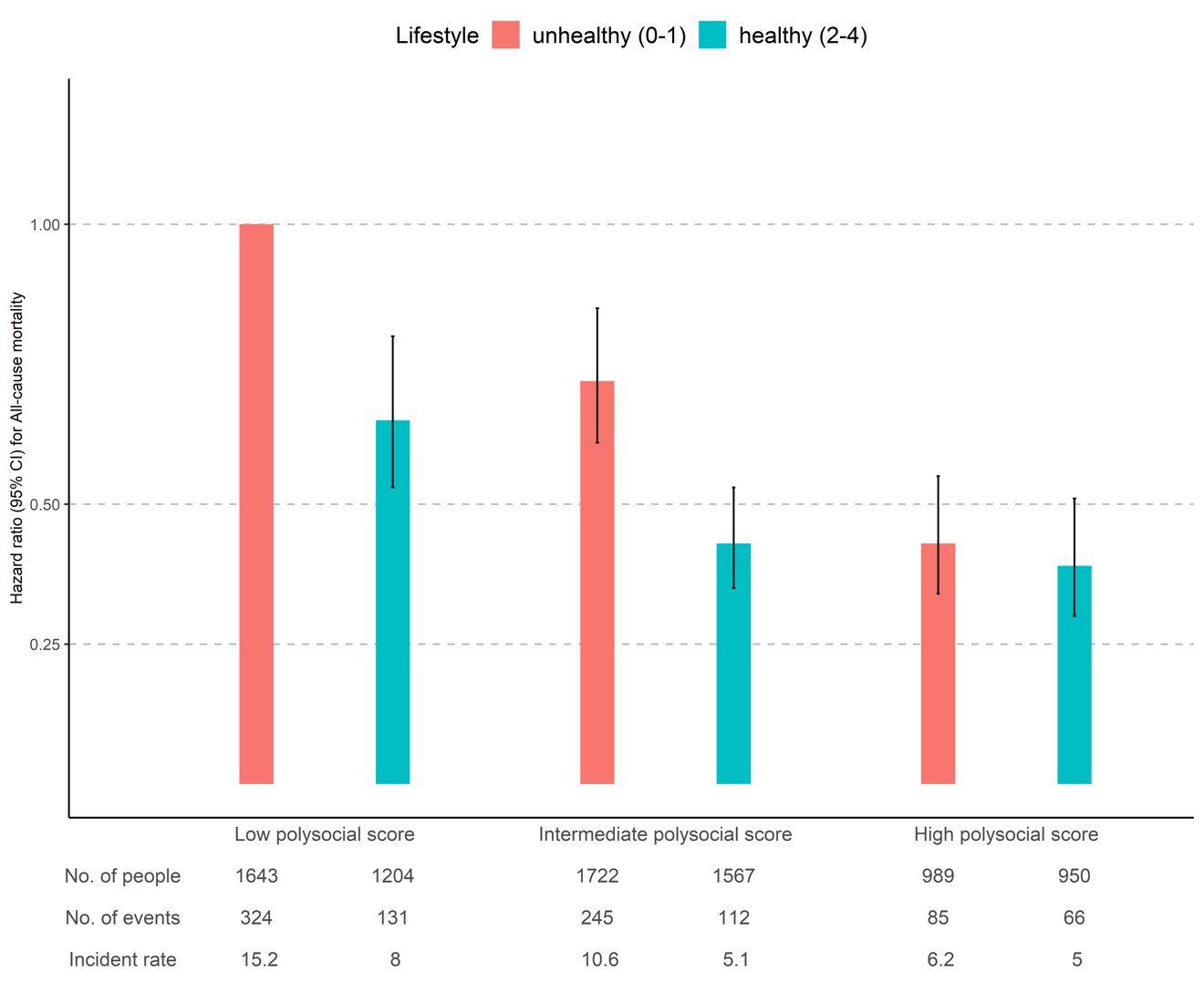


Note: CI, Confidence Interval. Hazard ratios were adjusted for sex and ethnicity (Whites & Others). For the composite healthy lifestyle score, the unhealthy level represented 0 and 1, and the healthy level represented 2,3, and 4.

**Supplementary Figure 12.** The joint effect of composite lifestyle score and polysocial score on all-cause mortality for participants who were frail and male (n=5,822).


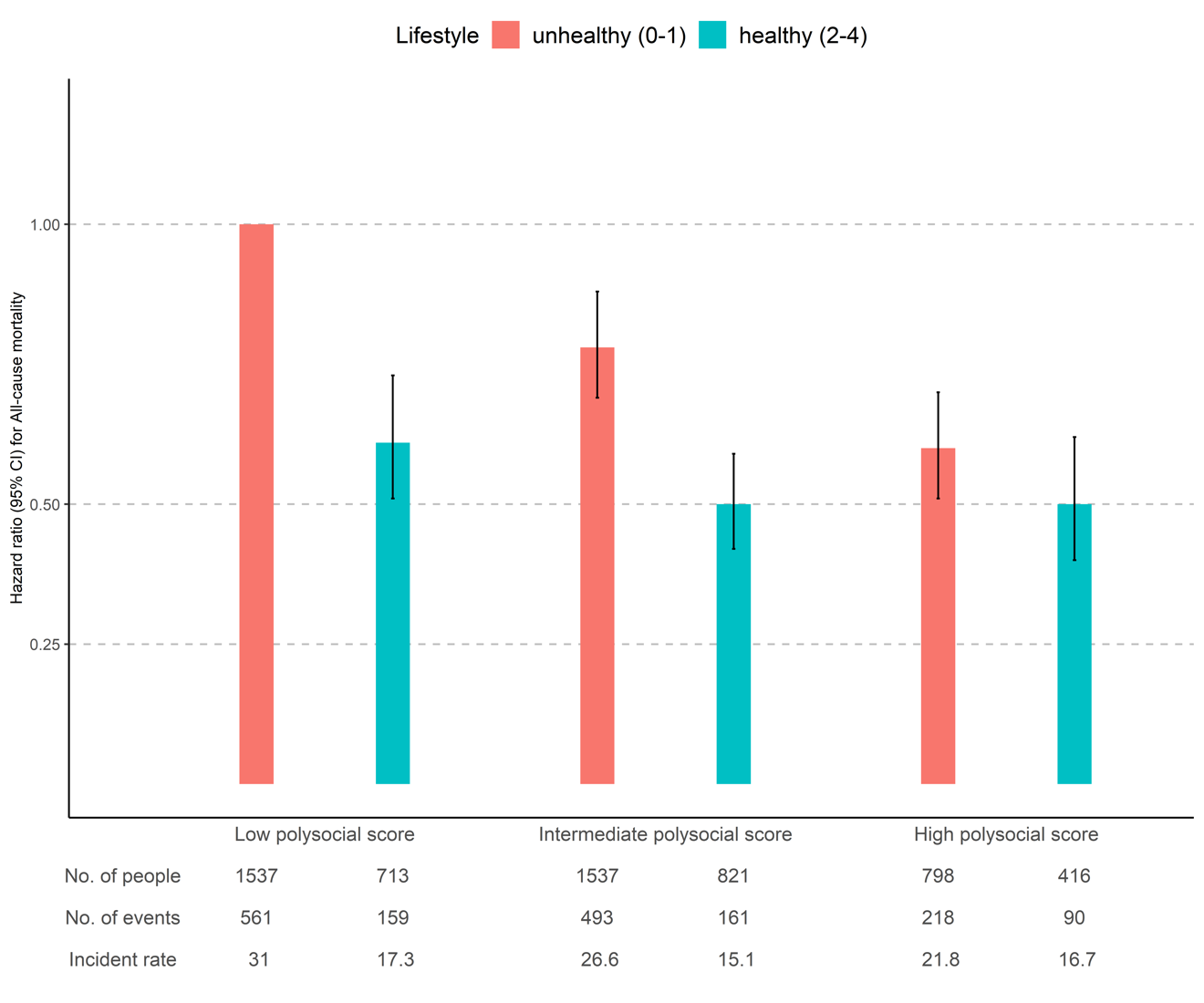
Note: CI, Confidence Interval. Hazard ratios were adjusted for age and ethnicity (Whites & Others). For the composite healthy lifestyle score, the unhealthy level represented 0 and 1, and the healthy level represented 2,3, and 4.

**Supplementary Figure 13.** The joint effect of composite lifestyle score and polysocial score on all-cause mortality for participants who were frail and female (n=9,722).


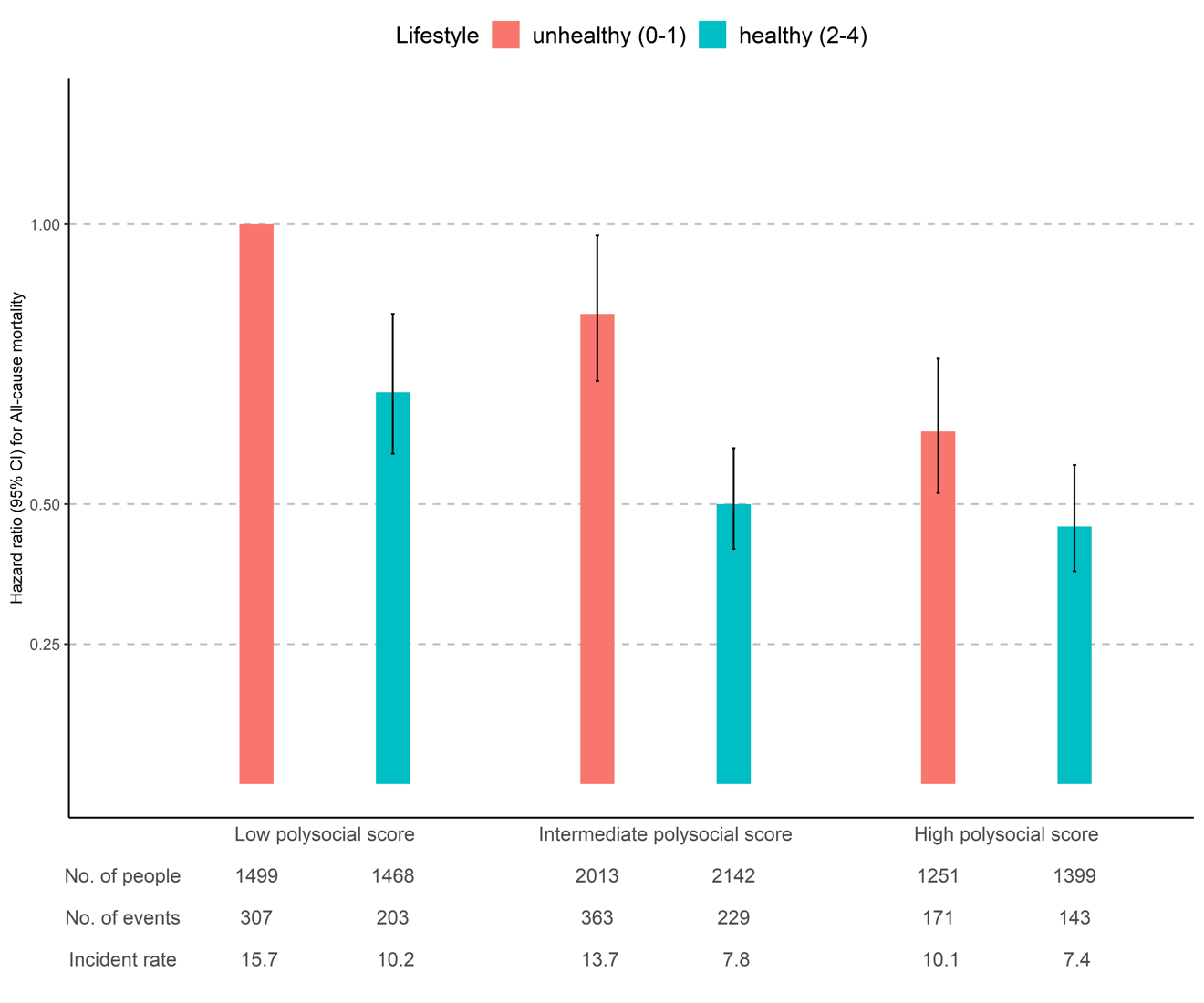
Note: CI, Confidence Interval. Hazard ratios were adjusted for age and ethnicity (Whites & Others). For the composite healthy lifestyle score, the unhealthy level represented 0 and 1, and the healthy level represented 2,3, and 4.
